# Vitamin D in Head and Neck Cancer: a Systematic Review

**DOI:** 10.1007/s11912-020-00996-7

**Published:** 2020-11-20

**Authors:** Antti Mäkitie, Iida Tuokkola, Göran Laurell, Outi Mäkitie, Kerry Olsen, Robert P. Takes, Ewa Florek, Krzysztof Szyfter, Cornelis F. M. Sier, Alfio Ferlito

**Affiliations:** 1 Department of Otorhinolaryngology—Head and Neck Surgery, Helsinki University Hospital, University of Helsinki, P. O. Box 263, FI-00029 HUS Helsinki, Finland; 2 Research Program in Systems Oncology, Faculty of Medicine, University of Helsinki, Helsinki, Finland; 3 Division of Ear, Nose and Throat Diseases, Department of Clinical Sciences, Intervention and Technology, Karolinska Institutet and Karolinska Hospital, Stockholm, Sweden; 4 Department of Surgical Sciences, ENT, Uppsala University, SE-75185 Uppsala, Sweden; 5 Children’s Hospital, University of Helsinki and Helsinki University Hospital, Helsinki, Finland; 6 Department of Otorhinolaryngology, Mayo Clinic, Rochester, MN USA; 7 Department of Otolaryngology—Head and Neck Surgery, Radboud University Medical Center, Nijmegen, The Netherlands; 8 Laboratory of Environmental Research, Department of Toxicology, Poznan University of Medical Sciences, Poznan, Poland; 9 Institute of Human Genetics, Polish Academy of Sciences, Poznan, Poland; 10 Department of Surgery, Leiden University Medical Center, Leiden, The Netherlands; 11 Coordinator of International Head and Neck Scientific Group, Padua, Italy

**Keywords:** Carcinoma, Etiology, Deficiency, Malignancy, Surgery, Radiotherapy

## Abstract

**Purpose of review:**

Observational studies have shown that serum 25-OH vitamin D [25(OH)D] is inversely associated with overall cancer risk in many malignancies. We performed a systematic literature review to determine whether vitamin D deficiency is related to head and neck cancer (HNC) etiology and outcome.

**Recent findings:**

The search yielded five prospective studies reporting 25(OH)D levels prior to cancer diagnosis and their effect on the risk of HNC. Eight studies were cross-sectional or case-control studies, in which 25(OH)D levels were only measured after cancer diagnosis. Two studies found an inverse association between 25(OH)D level and HNC risk, while two other prospective cohort studies demonstrated no connection between 25(OH)D and HNC risk. Several studies reported cancer patients to have significantly lower 25(OH)D levels than controls. Associations between 25(OH)D and prognosis and mortality were variable.

**Summary:**

The link between vitamin D and HNC has so far only been investigated in a few observational, prospective, and case-control studies. Vitamin D deficiency may be more common in HNC patients than in the healthy population. There is no evidence for a causal relationship. Further studies are needed to evaluate whether low 25(OH)D concentrations play a role in the development or outcome of HNCs.

**Supplementary Information:**

The online version contains supplementary material available at 10.1007/s11912-020-00996-7.

## Introduction

The recent GLOBOCAN report on cancer burden worldwide estimates that there is a steady increase in the incidence of head and neck cancer (HNC) [1]. Besides the well-known causative factors such as smoking, heavy drinking, and human papillomavirus behind this increase, several others have been suggested but remain inadequately investigated [2]. More research is therefore needed to identify novel preventive and treatment measures [3].

Recent research has suggested a role for vitamin D in HNC. Ingested vitamin D undergoes two hydroxylation steps to become the active metabolite. Pre-vitamin D is also synthesized in the skin from dehydrocholesterol by UV radiation. Vitamin D is then converted in the liver to 25-hydroxyvitamin-D (25(OH)D), or calcifediol. This precursor of active vitamin D undergoes another hydroxylation step in the kidneys to become the active form of vitamin D, 1alpha-25-dihydroxyvitamin D (1,25(OH)_2_D), or calcitriol. Calcitriol is the hormonal form of vitamin D and functions systemically via the vitamin D receptor (VDR), present in various tissues and cells. Vitamin D has long been recognized as having an important role in the regulation of calcium homeostasis and in maintaining bone health. Active vitamin D promotes calcium absorption from the digestive tract and reabsorption in the kidneys. Calcium is released from the bones through parathyroid hormone regulation. In recent decades, however, both VDRs and 1alpha-hydroxylase have been found in several other tissues beyond those traditionally associated with vitamin D metabolism, suggesting a more versatile role in whole body homeostasis [4••].

Some studies have explored a possible cause and effect relationship between 25(OH)D levels and disease risk. For example, a correlation between 25(OH)D levels and myocardial infarction has been found. Vitamin D has also been suggested as a possible treatment for autoimmune diseases due to its ability to block inflammation mediated by T cells. In addition, vitamin D derivatives have been successfully used to treat certain skin diseases such as psoriasis and acne [5, 6].

In mouse and hamster studies, vitamin D has been found to reduce the growth and development of HNCs [7–9]. In vitro studies show that the effects are mediated through several mechanisms. These include inhibiting proliferation and angiogenesis, as well as potentially reducing metastasizing. Inhibition of proliferation appears to be based on cell cycle arrest in the G0/G1 phase [10–12] by vitamin D or its derivatives by activating expression of the cell cycle-regulating inhibitor proteins p21 and p27 [13–15]. Inhibition of angiogenesis is suspected to be due to decreased HBp17/FGFBP-1 protein expression caused by 1,25(OH)_2_D or its derivatives. This will in turn lower the levels of the angiogenic fibroblast growth factor-2 (FGF-2) [16, 17]. In one study, 1,25(OH)_2_D reduced the production of angiogenic growth factors VEGF and PDGF in oral cancer cells [12]. An effect on metastatic potential was revealed in only one study, which found that the vitamin D analogue MART-10 inhibited head and neck squamous cell carcinoma (HNSCC) cell migration and invasion into other tissues and reduced epithelial–mesenchymal transition by inhibiting Snail, Twist, and MMP-9 expression [18].

Observational studies have demonstrated that 25(OH)D level is inversely associated with overall cancer risk and the risk of intestinal cancer [19, 20], but not prostate cancer [21]. Based on other cancer studies, low 25(OH)D concentrations may be associated with a greater risk of HNC. Duffy et al. noted in preclinical studies that calcitriol enhanced the effect of certain cytotoxic agents in cancer cells. Vitamin D supplementation increased disease-free survival in patients with breast cancer or colon cancer. A higher 25(OH)D level was associated with a better prognosis [22]. These observations suggest that vitamin D supplementation may have an effect on treatment or prognosis. However, a recent review on meta-analyses on observational studies reported that compelling evidence showing that vitamin D supplementation effectively improves survival of patients with cancer remains lacking [23].

In this literature review, we have focused on studies evaluating vitamin D as an etiological and prognostic factor for HNCs. We wanted to better understand the role of vitamin D in HNC by analyzing epidemiological studies regarding 25(OH)D levels in this patient population. Our hypothesis was that low 25(OH)D level is a predisposing factor in HNC. In addition, we searched for studies evaluating the effect of low 25(OH)D levels on cancer treatment outcomes, complications, and risk of cancer recurrence.

## Materials and Methods

This study followed the 2009 PRISMA criteria (Preferred Reporting Items for Systematic Reviews and Meta-Analysis) applied to a systematic review [24].

### Inclusion Criteria

The first inclusion criterion was that the selected study had to include people with HNC. Studies comprising participants with other cancers were included if separate results were available for HNC. The inclusion criterion for vitamin D was that serum 25(OH)D levels were measured. The literature review was limited to English-language publications. The review includes studies using the same material with different endpoints. Inclusion criteria did not, however, require certain endpoints from the included studies. The studies needed to have assessed the connection between 25(OH)D levels and HNC or compared 25(OH)D levels between HNC patients and a control population.

### Exclusion Criteria

We excluded from the systematic review studies that focused on the effect of vitamin D supplements on cancer treatment and studies which dealt with the connection between vitamin D and HNC at a cellular level or in animals.

### Search Methods

The systematic review was performed using the database systems of Ovid Medline, PubMed, and Scopus. Initially searches used several different terms to yield a limited but nevertheless comprehensive result.

First, we tested the effect of different search terms on the distribution of results, and based on the test queries (Appendix 1: searches 1–3, 6–7), we decided for the Medline search to use the MeSH terms “vitamin D” and “head and neck neoplasms.” From these, more precise MeSH terms were included: “squamous cell carcinoma of head and neck,” “mouth neoplasms,” “laryngeal neoplasms,” “nose neoplasms,” and “pharyngeal neoplasms.” The MeSH term “vitamin D” was extended to cover all the sub-terms, such as “cholecalciferol,” “ergocalciferol,” “calcitriol,” and “25-hydroxyvitamin D2.” In addition, we included search terms outside the MeSH terms: “head and neck” and “cancer,” “head and neck carcinoma,” and “vitamin D.”

Based on the practice searches in PubMed (Appendix 1, searches 4 and 9), we selected the MeSH major topic terms “head and neck neoplasms.” In addition, the MeSH term “vitamin D” was used in the search, which was extended to include “ergocalciferols.” For the Scopus search terms, we selected keywords with the proximity operators “head W/2 neck W/2 cancer” and “vitamin W/2 D.”

The final Medline search was performed on 17th of February 2020, the final PubMed search on 19th of February 2020, and the Scopus search on 10th of February 2020. For clarity we decided to only examine studies that measured the subjects’ 25(OH)D levels.

### Data Collection

From the selected studies, we collected information on the method for measuring vitamin D, sample size, subjects’ cancer, nationality, ethnicity of both the subjects and control group, and subject endpoints. The reported results were recorded as an odds ratio (OR), relative risk (RR), or hazard ratio (HR), if they had been specified. If the above ratios were not specified, results were reported in values, for example, the median vitamin D levels or vitamin D deficiency rate.

### Assessment of the Sources of Error

The sources of error of selected studies were assessed using a model developed by Viswanathan et al., the Item Bank for Assessing Risk of Bias and Confounding for Observational Studies of Interventions or Exposures [25].

## Results

### Selected Studies

**Fig. 1:**
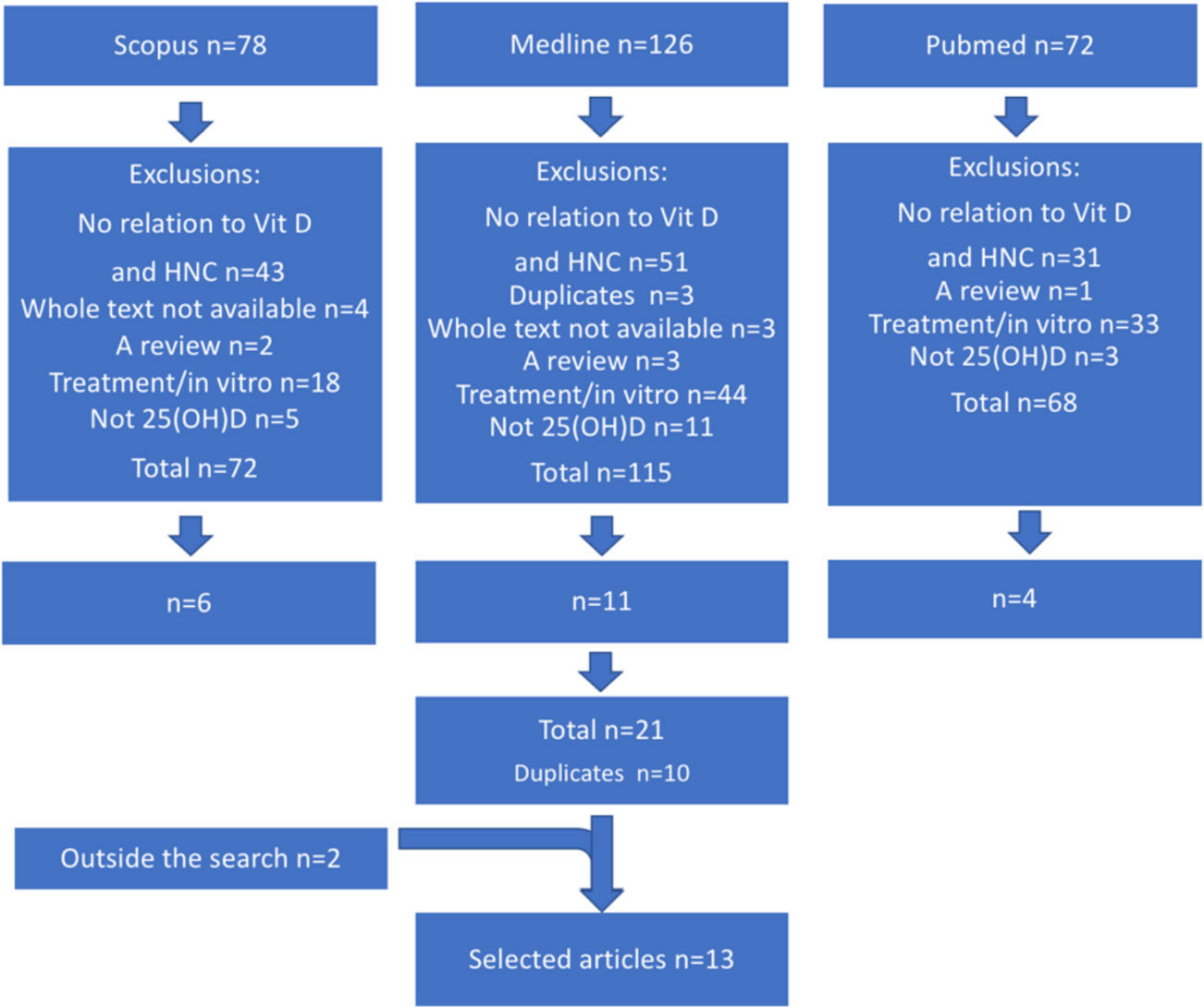
Flow chart for the search (HNC = head and neck cancer)

We examined a total of 276 studies, and after the inclusion and exclusion criteria were applied and duplicates removed, 13 studies remained (Fig. 1). In addition to these 13 studies, 13 studies investigated the connection between vitamin D and HNC, but they had to be excluded as they did not measure 25(OH)D. Details of these studies are summarized in Appendix 2.

Based on the assessment of source error, we detected a risk of non-participation bias in five out of the 13 included studies, when the most unwell or at-risk individuals did not participate the study [26•, 27, 28, 29•, 30•]. In three of the studies, there was a risk that this reduction in participation may result in bias due to small sample size [31–33]. The source of error assessment suggested that in six studies there was a risk of confounding bias as they did not sufficiently incorporate confounding factors. In their analyses, these studies did not take into account the seasonal variation in the participants’ 25(OH)D levels or sun exposure [32–37]. In six studies, the subjects’ alcohol consumption was not considered, and five studies did not consider the subjects’ smoking habits [32–34, 36, 37].

Table 1 summarizes the studies found in the review and the data collected from them. The search yielded five prospective studies [26•, 27, 28, 29•, 30•] reporting data on measured 25(OH)D levels prior to cancer diagnosis and the effect of these levels on the risk of HNC. The other eight studies were cross-sectional or case-control studies, in which 25(OH)D levels were only measured after cancer diagnosis.

**Table 1:** Thirteen selected studies evaluating vitamin D levels in HNC patients

Authors	Study design	Vitamin D measure	*n*	Subjects and controls	Endpoint	Direction of effect	Result HR/RR/OR (95% CI)
Weinstein et al. 2018 [30•]	Cohort, prospective	25(OH)D	398	HNSCC (oral, pharynx, and larynx), smoking men (ATBC), Finns	CA deaths	−	25(OH)D upper vs lower quintile OR = 0.74 (0.42–1.30)
Skaaby et al. 2014 [28]	Cohort, prospective	25(OH)D	44	HNC, three different cohorts (Monica10, Inter99, Health2006), Danes	CA risk	−	10 nmol/l 25(OH)D increase in OR = 0.97 (0.84–1.12) *p* = 0.69
Arem et al. 2011 [29•]	Nested case-control, prospective	25(OH)D	340 vs 340	HNCSCC (oral, pharynx, and larynx) and healthy smoking men (ATBC), Finns	CA risk	−	25(OH)D < 25 vs 50–75 nmol/l OR = 0.96 (0.58–1.59) *p* = 0.65 Lowest vs highest quartile of 25(OH)D OR = 1.02 (0.62 to 1.69)
Meyer et al. 2011 [38]	Cross-sectional	FFQ 25(OH)D	522	HNC, Canadians	Risk of recurrence Risk of secondary CA Mortality	−	HR = 1.12 (0.63–1.74) HR = 0.72 (0.40–1.30) HR = 0.85 (0.57–1.28)
Bochen et al. 2018 [36]	Cross-sectional	25(OH)D	231 vs 232	HNSCC and healthy, Germans	Survival Immune cells, NK cell activity	+	HNC: lower 25(OH)D *p* < 0.0001. Lower 25(OH)D: shorter survival times, *p* = 0.0085. Higher 25(OH)D: increased tumor immune cells
Nejatinamini et al. 2018 [33]	Cross-sectional	FFQ (3 days) 25(OH)D pre- and post-treatment	28	Awaiting HNC radiotherapy therapy, Canadians	Mucositis and muscle loss level	+	Mucositis 25(OH)D mean ± SD 47.2 ± 17.9 vs 62.3 ± 14.0 nmol/L, *p* = 0.025 multiple regression analysis of 25(OH)D muscle mass *B* = 0.74, SE = 0.36, *p* = 0.01, *B* = 0.63, SE = 0.37, *p* = 0.02
Anand et al. 2017 [34]	Cross-sectional	VDR expression 25(OH)D	110 vs 95	OSCC and premalignant lesions and healthy, Indians	–	+	In cancer, significantly lower 25(OH)D, D-score ka − 1.90 vs − 1.33 *p* = 0.002
Fanidi et al. 2016 [26•]	Nested case-control, prospective	25(OH)D	350 vs 940	HNC (oral, oropharynx, pharynx, larynx, and others), European cohort (EPIC)	CA risk Mortality	+	OR(log2) = 0.77 (0.56–0.88) *p* = 0.001 HR = 0.73 (0.55–0.97)
Mostafa et al. 2016 [35]	Cross-sectional	25(OH)D	50 vs 30	HNSCC and healthy, Egyptians	–	+	25(OH)D medians 40.35 vs 118.75 nmol/l, *p* < 0.001
Grimm et al. 2015 [32]	Cross-sectional case-control	VDR expression 25(OH)D	42 vs 46 vs 5	OSCC, precursors (SIN I to III), and healthy, Germans	–	+	62% vit D < 12,5 ng/ml, 38% vit D < 25 ng/ml
Afzal et al. 2013 [27]	Cohort, prospective	25(OH)D	122 vs 400	HNC, Danes	Tobacco-related CA risk	+	50% 25(OH)D decrease, HR = 1.44 (1.19–1.73)
Orell et al. 2012 [31]	Cross-sectional	25(OH)D	65	HNSCC, compared with the general population level, Finns	–	+	In cancer, vitamin D deficiency, 25(OH)D < 50 nmol/l 65.6% vs 21.3% *p* < 0.0001
Gugatschka et al. 2011 [37]	Case-control	25(OH)D	88 vs 88	HNSCC recently diagnosed in Europe?	Disease-free survival, overall survival	+	RR = 0.85 (0.75–0.96) *p* = 0.01, RR = 0.89 (0.83–0.97) *p* = 0.006

*FFQ* food frequency questionnaire, *HNC* head and neck cancer, *HNSCC* head and neck squamous cell carcinoma, *OSCC* oral squamous cell carcinoma, *VDR* vitamin D receptor

Five studies focused on Nordic populations, four on European populations, two on North American populations, one on an Indian, and one on an Egyptian population (Table 1).

In three studies, the primary endpoint was overall occurrence of cancer. HNC risk was therefore not a specified endpoint, but the studies were included as they provided separate data regarding HNCs. In four studies, the endpoint was HNC risk, for three studies it was mortality, and in two studies, survival time. Two studies had an endpoint related to cancer treatment outcome, one on mucositis and muscle loss and the other on risk of recurrence and risk of secondary cancer. In four studies, the endpoint could not be determined, because the only analyses they contained were comparisons of median measured levels of 25(OH)D or vitamin D deficiency status between different groups (Table 1).

### Cancer Risk

In terms of cancer risk, two studies found an inverse association between 25(OH)D level and HNC risk [26•, 27]. In the EPIC cohort study, a case-control study involving 350 HNC patients and individually matched controls, Fanidi et al. [26•] found that doubling of 25(OH)D plasma levels significantly reduced the cancer risk in the HNC patients compared with the controls. In addition, Afzal et al. [27] stated in their Danish prospective cohort study including 122 HNC patients that a 50% decrease in 25(OH)D plasma levels significantly increased cancer risk. In contrast, two prospective cohort studies by Skaaby et al. [28] and Arem et al. [29•] demonstrated no connection between 25(OH)D and HNC risk.

In several studies, cancer patients were found to have significantly lower 25(OH)D levels than controls. Anand et al. [34] noted the same for oral cancer patients that Mostafa et al. [35] and Bochen et al. [36] observed for head and neck squamous cell carcinoma patients in general: all these patient groups had significantly lower 25(OH)D levels as compared with healthy controls. Orell-Kotikangas et al. [31] demonstrated in a series of 65 HNSCC patients that vitamin D deficiency was significantly more prevalent in these patients than in the Finnish population in general. Similarly, Grimm et al. found in all 42 oral cancer patients either moderate or severe vitamin D deficiency [32].

### Post-treatment Survival and Mortality

Weinstein et al. [30•] found in a prospective cohort study comprising 398 male smokers in Finland no connection between 25(OH)D levels and HNC mortality. Similarly, Meyer et al. [38] did not find an association between 25(OH)D levels and mortality in 522 Canadian HNC patients. Fanidi et al. [26•] found an inverse association between 25(OH)D levels and mortality in HNC patients; however, when the stage of cancer was taken into account, the association was significantly weaker.

Both Bochen et al. [36] and Gugatschka et al. [37] found higher 25(OH)D levels prior to treatment to be associated with longer post-treatment survival. In the latter study, the authors also observed an association with longer disease-free survival. Bochen et al. found that higher 25(OH)D levels were associated with higher peri-tumoral immune cell infiltration [36].

### Cancer Complications and Risk of Recurrence

A study on complications in cancer treatment found that in Canadian HNC patients, lower 25(OH)D levels were associated with more serious mucositis and lower muscle mass [33]. Meyer et al. did not find a relationship between 25(OH)D levels and risk of HNC recurrence or the occurrence of secondary cancer [38].

## Discussion

Systematic reviews of the relationship between vitamin D and HNC have not been conducted previously. The present review revealed that the results from the studies were varying, showing evidence of both for and against an association between the risk of HNC and level of 25(OH)D. However, in all five studies that compared 25(OH)D levels between HNC patients and healthy individuals, HNC patients were found to have much lower levels of vitamin D. In two out of three studies, 25(OH)D level was not observed to have an effect on mortality. However, higher 25(OH)D levels were observed to have a protective effect on survival.

The studies show that vitamin D deficiency may be more common in HNC patients than in the healthy population. There is no evidence for a proven causal relationship. Cancer patients’ lower 25(OH)D levels could be partially explained by the eating and swallowing difficulties caused by the cancer and its treatment. As a result, dietary intake of vitamin D may be reduced.

When vitamin D was observed to have an inverse association with HNC risk, this was then also correlated with smoking. Afzal et al. focused on tobacco-related cancer risk [27], and Fanidi et al. found an association only in smokers or ex-smokers [26•]. Afzal et al. found 25(OH)D levels to be associated only with smoking-related cancers, such as lung, head and neck, bladder, kidney, liver, and esophageal cancers, but not with other cancers [27]. The protective effect of high vitamin D might only be associated with HNC cases related to smoking.

We observed in the included studies several potential sources of bias as some patient groups did not participate the study, the sample size in the series was small, or the confounding factors were not sufficiently incorporated. Further, in a significant number of studies, the main circulating vitamin D metabolite 25(OH)D was not measured, and the studies failed to fulfill our inclusion criteria.

Meta-analyses and randomized controlled trials (RCT) on the effect of vitamin D on cancer risk in general have so far yielded contradictory results. In a RCT of postmenopausal women, vitamin D + calcium supplement decreased their all-cancer risk [39]. However, another RCT found no association between vitamin D and invasive cancer risk [40]. Based on meta-analyses, however, vitamin D supplementation does appear to be associated with lower total cancer mortality [41, 42]. Keum et al. found that it did not have an effect on total cancer incidence [42]. More research is needed, with sufficiently long follow-up and assessment of the subject’s baseline 25(OH)D levels.

The role of vitamin D in HNC management has been largely ignored. Studies to date have not identified a clear clinical effect on vitamin D treatment outcome. The best evidence for the benefits of vitamin D in cancer treatment would be provided by a RCT. However, such studies have not been conducted for HNCs. They would require long follow-up periods, which affect subject compliance. Also, there is uncertainty as to the optimal dose of vitamin D for cancer treatment or prevention.

The existing studies regarding administration of vitamin D have, for example, observed a stimulating effect on the immune system of HNC patients. Walsh et al. observed that a 1,25(OH)_2_D supplement given prior to surgery increased CD4 and CD8 cell levels for 3 weeks and increased CD69 expression, suggesting T cell activation. They also found 1,25(OH)_2_D treatment to be effective in prolonging the time to recurrence of cancer. The results were not statistically significant due to the small sample size [43]. Lathers et al. showed that 25(OH)D supplements improved the immune response of HNC patients by temporarily reducing the number of CD34-positive cells and increasing the number of IL-12 and IFN-γ-neurotransmitters [44]. Walker et al. observed that giving 1,25(OH)_2_D treatment prior to surgery increases the plasma levels of both type 1 and type 2 T helper cell system mediators. Giving 1,25(OH)_2_D supplement also increased the levels of the tumorigenic and angiogenic mediators IL-8 and VEGF in plasma, but not in cancerous tissue [45].

The strength of our review is a comprehensive systematic literature search performed according to strict criteria. Results which both strengthen and weaken the association were observed, so the risk of reporting bias is low.

## Conclusions

This review revealed that the link between vitamin D and HNCs has so far only been investigated in observational and case-control studies. Based on current knowledge, it is not possible to definitively deduce a causal connection between HNCs and vitamin D. Our results suggest an inverse relationship between the risk of HNC and 25(OH)D level. Further, there may be a direct relationship between 25(OH)D levels and both overall and disease-specific HNC survival. However, there has not been a single RCT on the efficacy of vitamin D supplements as a preventive measure against HNC occurrence or on treatment outcome. Such RCTs are needed to provide more data and to guide in vitamin D supplementation recommendations for patients with HNC.

## Supplementary Information

ESM 1 (PDF 131 kb).

## Data Availability

Not applicable.
