# Supplementary material for: Vitamin D in Head and Neck Cancer: a Systematic Review
Source: Curr Oncol Rep. 2020 Nov 20;23(1):5. doi: 10.1007/s11912-020-00996-7 (PMC7679336; doi:10.1007/s11912-020-00996-7)
Supplement: Supplementary file 1 — (PDF 131 kb). [file 11912_2020_996_MOESM1_ESM.pdf]

Vitamin D in Head and Neck Cancer: A Systematic Review

Current Oncology Reports

Antti Mäkitie[1,2,3], Iida Tuokkola[1], Göran Laurell[4], Outi Mäkitie[5], Kerry Olsen[6], Robert P. Takes[7], Ewa Florek[8], Krzysztof Szyfter[9], Cornelis F.M. Sier[10], Alfio Ferlito[11]

Corresponding author:

Antti A. Mäkitie MD PhD

Department of Otorhinolaryngology - Head and Neck Surgery

University of Helsinki and Helsinki University Hospital

P.O.Box 263. FI-00029 HUS, Helsinki, Finland

Email: antti.makitie@helsinki.fi

Tel.: +358-50-4286847

Appendix 1: Search methods

Search 1: Ovid Medline 16.1. keywords

1 *"head and neck neoplasms"/ or exp "squamous cell carcinoma of head and neck"/ or exp mouth neoplasms/ or exp laryngeal neoplasms/ or exp nose neoplasms/ or exp pharyngeal neoplasms/
2 (Head and neck cancer and) .mp. [mp=title, abstract, original title, name of substance word, subject heading word, floating sub-heading word, keyword heading word, organism supplementary concept word, protocol supplementary concept word, rare disease supplementary concept word, unique identifier, synonyms]
3 (head and neck and carcinoma).mp. [mp=title, abstract, original title, name of substance word, subject heading word, floating sub-heading word, keyword heading word, organism supplementary concept word, protocol supplementary concept word, rare disease supplementary concept word, unique identifier, synonyms]
4 1 or 2 or 3
5 exp *Vitamin D/ or vitamin D.mp.
6 4 and 5

135 results → 2 duplicates, 56 excluded → 77 remaining

Search 2. Medline 17.1. keywords

1. *"head and neck neoplasms"/ or exp "squamous cell carcinoma of head and neck"/ or exp mouth neoplasms/ or exp laryngeal neoplasms/ or exp nose neoplasms/ or exp pharyngeal neoplasms/
2. (head adj2 neck adj3 cancer).mp. [mp=title, abstract, original title, name of substance word, subject heading word, floating sub-heading word, keyword heading word,

organism supplementary concept word, protocol supplementary concept word, rare disease supplementary concept word, unique identifier, synonyms]

3. (head adj3 neck adj3 carcinoma).mp. [mp=title, abstract, original title, name of substance word, subject heading word, floating sub-heading word, keyword heading word, organism supplementary concept word, protocol supplementary concept word, rare disease supplementary concept word, unique identifier, synonyms]
4. 1 or 2 or 3
5. exp *Vitamin D/ or vitamin D.mp.
6. 4 and 5
7. exp Therapeutics/
8. exp Vitamin D Deficiency/
9. complicat*.mp.
10. exp Survival/ or surviv*.mp. or exp Survival Rate/
11. exp Early Medical Intervention/ or intervention*.mp.
12. Dietary Supplements/
13. supplement*.mp.
14. exp Conservative Treatment/ or treatment*.mp. or exp Treatment Outcome/ or exp Treatment Failure/
15. 7 or 8 or 9 or 10 or 11 or 12 or 13 or 14
16. 6 and 15

83 results, 2 duplicates, 32 excluded 32 → 49 remaining (same as search 1)

Search 3. Medline 29.1. keywords

1. vitamin D AND head and neck and cancer or carcinoma or neoplasm
2. limit one to five stars
3. *"head and neck neoplasms"/ or exp *"squamous cell carcinoma of head and neck"/ or exp mouth neoplasms/ or exp laryngeal neoplasms/ or exp nose neoplasms/ or exp pharyngeal neoplasms/
4. (head adj3 neck adj3 cancer).mp. [mp=title, abstract, original title, name of substance word, subject heading word, floating sub-heading word, keyword heading word, organism supplementary concept word, protocol supplementary concept word, rare disease supplementary concept word, unique identifier, synonyms]
5. (head adj3 neck adj3 carcinoma).mp. [mp=title, abstract, original title, name of substance word, subject heading word, floating sub-heading word, keyword heading word, organism supplementary concept word, protocol supplementary concept word, rare disease supplementary concept word, unique identifier, synonyms]
6. (head adj2 neck adj3 neoplasm).mp. [mp=title, abstract, original title, name of substance word, subject heading word, floating sub-heading word, keyword heading word, organism supplementary concept word, protocol supplementary concept word, rare disease supplementary concept word, unique identifier, synonyms]
7. 3 or 4 or 5 or 6
8. exp *Vitamin D/ or vitamin D.mp.
9. 7 and 8
10. 2 or 9
11. 10 and "Humans".sa_suba.
12. limit 11 to English language

94 results, 2 duplicates, 69 excluded → 23 remaining (same as search 2, all the same as search 1)

Search 4. PubMed 3.2. keywords
(("head and neck neoplasms"[MeSH Terms] OR ("head"[All Fields] AND "neck"[All Fields] AND "neoplasms"[All Fields]) OR "head and neck neoplasms"[All Fields] OR ("head"[All Fields] AND "neck"[All Fields] AND "cancer"[All Fields]) OR "head neck cancer"[All Fields]) AND ("vitamin d"[MeSH Terms] OR "vitamin d"[All Fields] OR "ergocalciferols"[MeSH Terms] OR "ergocalciferols"[All Fields])) AND ("epidemiology"[Subheading] OR "epidemiology"[All Fields] OR "epidemiology"[MeSH Terms])
limits English, humans

97 results, 92 duplicates → 5 remaining (same as search 3) 0 new

Search 3. Scopus 10.2. keywords KEY (Head W/2 neck W/2 cancer) AND KEY (vitamin W/2 d), limit English
78 results, 72 duplicates → 6 remaining (same as searches 1 and 3)

Search 6. Ovid Medline 12.2. keywords as in Search 1, limits English and full text
55 results, 45 duplicates → 10 remaining (same as searches 3 and 1)

Search 7. Ovid Medline 14.2. keywords as in Search 2, limits English
76 results, 2 duplicates, 60 excluded → 14 remaining (same as search 3, all the same as search 2)

Search 8. Ovid Medline 17.2. keywords as in Search 1, limits English
126 results, 3 duplicates, 112 excluded → 11 remaining (same as search 1)

Search 9. PubMed 19.2. keywords
(("head and neck neoplasms"[MeSH Terms] OR ("head"[All Fields] AND "neck"[All Fields] AND "neoplasms"[All Fields]) OR "head and neck neoplasms"[All Fields] OR ("head"[All Fields] AND "neck"[All Fields] AND "cancer"[All Fields]) OR "head neck cancer"[All Fields]) AND ("vitamin d"[MeSH Terms] OR "vitamin d"[All Fields] OR "ergocalciferols"[MeSH Terms] OR "ergocalciferols"[All Fields])) AND ((("epidemiology"[MeSH Major Topic] OR "vitamin d deficiency"[MeSH Major Topic]) OR "therapeutics"[MeSH Major Topic]) OR "survival"[MeSH Major Topic]) OR "dietary supplements"[MeSH Major Topic]
limits English

116 results, 112 duplicates → 4 remaining (same as search 8)

Search 10. PubMed 19.2. keywords
("head and neck neoplasms"[MeSH Major Topic] NOT (("thyroid neoplasms"[MeSH Terms] OR ("thyroid"[All Fields] AND "neoplasms"[All Fields]) OR "thyroid neoplasms"[All Fields]) OR "esophageal neoplasms"[MeSH Major Topic])) AND ("vitamin d"[MeSH Major Topic] OR "ergocalciferols"[MeSH Major Topic])
limits English

72 results, 68 duplicates → 4 remaining (same as search 8)

In addition, the sources of the reviews in the search results included 4 articles outside the actual systematic review search.

Appendix 2.

Results outside the search criteria

**Study authors**	**Measuring vitamin D (concentration, food diary, polymorphism)**	**n**	**Subjects and controls**	**Endpoint**	**Result**
Giovannucci et al. 2006 [1]	Estimated using model 25(OH)D, prospective	51	Oral and throat cancer, USA,	Cancer risk	25 nmol/l<br>Vit D increase

Study authors	Measuring vitamin D (concentration, food diary, polymorphism)	n	Subjects and controls	Endpoint	Result
			cohort of 47,800 men		RR=0.30 (0.11-0.81)
Dudding et al. 2018 [2]	Risk deduced from 5 gene variants, describes serum 25(OH)D	5133 vs. 5984	Oral cavity and oropharyngeal cancers, North and South America and Europe	Cancer risk	OR=1.1 (0.74-1.40) p=0.93
Yokosawa et al. 2018 [3]	FFQ (diet and D suppl.)	434	HNC (oral, oropharynx, pharynx and larynx), USA	Cancer mortality, mortality, risk of recurrence	HR=0.79 (0.40-1.56) HR=0.84 (0.48-1.47) HR=0.57 (0.25-1.28) p=0.48
Lipworth et al. 2009 [4]	FFQ (2 yrs, little use of supplements)	804 vs. 2080	Oral and throat cancer patients, Italy	Cancer risk	Vit D upper vs. lower tertile OR=0.76 (0.60-0.94) p=0.012
Bidoli et al. 2003 [5]	FFQ (2yrs, no supplements)	527 vs. 1297	Cancer of the larynx, Italy and Switzerland	Cancer risk	Vit D upper vs lower quintile OR=1.8 (1.2-2.6)
Negri et al. 2000 [6]	FFQ (2yrs) no supplements	754 vs. 1775	Oral and throat cancer, Italy and Switzerland	Cancer risk	Controls by 1 SD Vit D increase OR=0.83 (0.67-1.04)

Study authors	Measuring vitamin D (concentration, food diary, polymorphism)	n	Subjects and controls	Endpoint	Result
Malodobra-Mazur et al. 2012 [7]	SNP polymorphisms in the VDR gene	73 vs. 100	Oral cancer, Poland	Cancer risk	rs2238135 G/C vs G/G OR=3.16 (1.67–5.96) p=0.0002
Zeljic et al. 2012 [8]	VDR and CYP polymorphisms	110 vs. 122	Oral cancer, Serbia, white controls, blood donors	Cancer risk, survival	CYP24A1 AG OR=0.281 (0.139–0.568), p=0.000 FoKI: ff poorer prognosis p = 0.012
Hama et al. 2011 [9]	VDR polymorphisms (Cdx2, FokI, BsmI, ApaI, and TaqI)	204	HNSCC, Japan	Progression-free survival	FokI T/T vs C/C+C/T HR=3.03 (1.62-5.67) p=0.001
Bektas-Kayhan et al. 2010 [10]	VDR TaqI	64 vs. 87	OSCC, Turkey	Cancer risk, prognosis (TNM)	Tt genotype higher risk of OR=1.39 (1.02–1.90) p=0.036 T-allele associated with tumour size, otherwise no effect
Liu et al. 2005 [11]	VDR TaqI and FokI polymorphisms	719 vs. 821	With HNSCC (oral, hypopharynx, pharynx, larynx) white, USA and	Cancer risk	2 t/f variant: OR=0.74 (0.54-1.03) 3 or 4 var: OR=0.55

Study authors	Measuring vitamin D (concentration, food diary, polymorphism)	n	Subjects and controls	Endpoint	Result
			healthy controls		(0.38-0.79) p=0.001<br>tt: OR=0.72 (0.53-0.98)<br>ff: OR=0.64 (0.47-0.87)
Azad et al. 2012 [12]	GSV in vitamin D metabolism genes: VDR, GC, CYP24A1, CYP27A1, CYP27B1 and CYP2R1	511	HNC, Quebec, Canada	Vit D level,<br>survival,<br>risk of secondary cancer	GC and D CYP2R1 associated with a lower vit D level.<br>CYP24A1 GvsA HR=1.23 (1.00-1.51) p=0.05<br>CYP2R1 TvsC HR=0.59 (0.43–0.81)<br>p=0.001
Huang et al. 2011 [13]	VDR FokI and BsmI	171 vs. 176	NPC Han Chinese population	Cancer risk	ff vs FF OR=1.0<br>(0.56-1.79)<br>bB+BB vs bb OR=0.87 (0.49-1.53)

FFQ = Food frequency questionnaire

GSV = Gene sequence variation

HNC = Head and Neck cancer

HNSCC = Head and Neck squamous cell carcinoma

NPC = Nasopharyngeal cancer

SNP = Single nucleotide polymorphism

VDR = Vitamin D receptor

Reference list for Appendix 2

[1]. Giovannucci E, Liu Y, Rimm EB, Hollis BW, Fuchs CS, Stampfer MJ, et al. Prospective study of predictors of vitamin D status and cancer incidence and mortality in men. J Natl Cancer Inst. 2006;98(7):451-9.

[2]. Dudding T, Johansson M, Thomas SJ, Brennan P, Martin RM, Timpson NJ. Assessing the causal association between 25-hydroxyvitamin D and the risk of oral and oropharyngeal cancer using Mendelian randomization. Int J Cancer. 2018;143(5):1029-36.

[3]. Yokosawa EB, Arthur AE, Rentschler KM, Wolf GT, Rozek LS, Mondul AM. Vitamin D intake and survival and recurrence in head and neck cancer patients. Laryngoscope. 2018;128(11):E371-E376.

[4]. Lipworth L, Rossi M, McLaughlin JK, Negri E, Talamini R, Levi F, et al. Dietary vitamin D and cancers of the oral cavity and esophagus. Ann Oncol. 2009;20(9):1576-81.

[5]. Bidoli E, Bosetti C, La Vecchia C, Levi F, Parpinel M, Talamini R, et al. Micronutrients and laryngeal cancer risk in Italy and Switzerland: a case-control study. Cancer Causes Control. 2003;14(5):477-84.

[6]. Negri E, Franceschi S, Bosetti C, Levi F, Conti E, Parpinel M, et al. Selected micronutrients and oral and pharyngeal cancer. Int J Cancer. 2000;86(1):122-7.

[7]. Malodobra-Mazur M, Paduch A, Lebioda A, Konopacka M, Rogolinski J, Szymczyk C, et al. VDR gene single nucleotide polymorphisms and their association with risk of oral cavity carcinoma. Acta Biochim Pol. 2012;59(4):627-30.

[8]. Zeljic K, Supic G, Stamenkovic Radak M, Jovic N, Kozomara R, Magic Z. Vitamin D receptor, CYP27B1 and CYP24A1 genes polymorphisms association with oral cancer risk and survival. J Oral Pathol Med. 2012;41(10):779-87.

[9]. Hama T, Norizoe C, Suga H, Mimura T, Kato T, Moriyama H, et al. Prognostic significance of vitamin D receptor polymorphisms in head and neck squamous cell carcinoma. PLoS ONE. 2011;6(12):e29634.

[10]. Bektas-Kayhan K, Unur M, Yaylim-Eraltan I, Ergen HA, Toptas B, Hafiz G, et al. Association of vitamin D receptor Taq I polymorphism and susceptibility to oral squamous cell carcinoma. In Vivo. 2010;24(5):755-9.

[11]. Liu Z, Calderon JI, Zhang Z, Sturgis EM, Spitz MR, Wei Q. Polymorphisms of vitamin D receptor gene protect against the risk of head and neck cancer. Pharmacogenet Genomics. 2005;15(3):159-65.

[12]. Azad AK, Bairati I, Qiu X, Huang H, Cheng D, Liu G, et al. Genetic sequence variants in vitamin D metabolism pathway genes, serum vitamin D level and outcome in head and neck cancer patients. Int J Cancer. 2013;132(11):2520-7.

[13]. Huang X, Cao Z, Zhang Z, Yang Y, Wang J, Fang D. No association between Vitamin D receptor gene polymorphisms and nasopharyngeal carcinoma in a Chinese Han population. Biosci Trends. 2011;5(3):99-103.
